# Discriminatory Attitudes Towards People Living With HIV Among Key Populations in Nigeria: A Latent Class Analysis

**DOI:** 10.1155/arat/1843342

**Published:** 2025-08-28

**Authors:** Babayemi O. Olakunde, Daniel A. Adeyinka, Chukwugozie Ujam, Ashenafi S. Cherkos, Hidayat B. Yahaya, Stanley Eneh, Chinwendu D. Ndukwe, James O. Anenih

**Affiliations:** ^1^Department of Population and Community Health, College of Public Health, University of North Texas Health Science Center, Fort Worth, Texas, USA; ^2^IVAN Research Institute, University of Nigeria, Nsukka, Enugu, Nigeria; ^3^Department of Research, Saskatchewan Health Authority, Saskatoon, Saskatchewan, Canada; ^4^National AIDS and STI Control Programme, Department of Public Health, Federal Ministry of Health, Abuja, Nigeria; ^5^Department of Community Prevention and Care Services, National Agency for the Control of AIDS, Abuja, Nigeria; ^6^Department of Policy, Planning, and Coordination, National Agency for the Control of AIDS, Abuja, Nigeria; ^7^African Institute of Health Policy and Health Systems, Abakaliki, Nigeria

**Keywords:** discrimination, key populations, people living with HIV, stigma

## Abstract

**Background:** HIV-related discrimination remains a significant barrier to the uptake of HIV prevention and treatment services in sub-Saharan Africa among key populations (KPs). However, despite the substantial risk of HIV among peers within their social networks, there is a paucity of data on their attitudes towards people living with HIV (PLHIV). This study aimed to examine discriminatory attitudes towards PLHIV among KPs in Nigeria.

**Methods:** This study was a secondary data analysis of the 2020 Integrated Biological and Behavioral Surveillance Survey in Nigeria, which included 17,975 KPs. We operationalized discriminatory attitudes as negative responses to questions on caring for PLHIV, buying food from PLHIV, working with PLHIV, sharing a meal with PLHIV, and a positive response to quarantining PLHIV. We conducted weighted descriptive statistics to summarize the data, and latent class analysis was used to determine the patterns of discriminatory attitudes. The predicted probabilities of the classes for each KP characteristic were estimated while holding all other characteristics in the model at their means. The data analysis was conducted using Stata 18.

**Results:** About 13.5% of participants indicated they would not provide care for PLHIV , 29.7% would not buy food from them, 15.8% would not work with them, 27.9% would not share a meal with them,and 16.3% believed that PLHIV should be quarantined. Three latent classes of discriminatory attitudes were identified: low discriminatory attitude (65%), moderate discriminatory attitude (23%), and high discriminatory attitude (11%). The highest predicted probability of high discriminatory attitude was observed among KPs with unknown HIV status (42%), followed by those residing in the Southeast region (39.7%).

**Conclusion:** Discriminatory attitudes towards PLHIV are common among KPs in Nigeria. Interventions aimed at reducing HIV-related discrimination should also target KPs.

## 1. Background

HIV-related discrimination refers to the unfair and unjust treatment of an individual based on their real or perceived HIV status [1]. It is one of the key manifestations of stigma [2] and poses a significant barrier to the uptake of HIV-related services and optimal health outcomes among people living with HIV (PLHIV) in sub-Saharan Africa (SSA) [3–5]. Discrimination against PLHIV can take various forms, including verbal and physical abuse, neglect, and denial of services or opportunities [6]. These acts of discrimination may be perpetrated by family members, intimate partners, co-workers, health workers, and community members [7, 8].

Several studies in SSA have reported a high prevalence of discriminatory attitudes towards PLHIV [9–11]. For example, a study conducted across 15 countries in SSA found that 47.1% of people held discriminatory attitudes towards PLHIV (would not buy vegetables from a vendor living with HIV or believed that children with HIV should not be allowed to attend school with those without HIV) [11]. Factors, such as limited knowledge of HIV, living in rural areas, lower educational and wealth levels, lack of mass media exposure, and no previous HIV testing, are associated with higher odds of having discriminatory attitudes towards PLHIV [9–11].

While there is an extensive body of literature on discriminatory attitudes towards PLHIV in SSA, most studies have predominately focused on the general population [12–14] and healthcare workers [15–18]. Despite their substantial HIV risk and active social networks, there is a paucity of data on discriminatory attitudes towards PLHIV among key populations (KPs) in SSA. Although KPs are often the victims, perpetration of HIV-related discrimination towards their peers living with HIV is not uncommon [19]. These negative attitudes are particularly significant in restrictive settings in SSA, where social networks play a crucial role in providing members with information, emotional, social, or financial support [20, 21]. There is growing evidence supporting the effectiveness and use of social network-based interventions for reaching KPs with HIV services [22–24]. However, such interventions may prove counterproductive in settings where discrimination against PLHIV is high within these networks.

Gaining insight into the attitudes of KPs towards PLHIV can inform the design of targeted interventions and enhance their engagements in HIV prevention and treatment services. Accordingly, this study examined discriminatory attitudes towards PLHIV among KPs in Nigeria.

## 2. Methods

### 2.1. Design and Data Source

This study was a secondary analysis of the 2020 Integrated Biological and Behavioral Surveillance Survey (IBBSS) conducted across 12 states in Nigeria [25]. The 2020 IBBSS survey included 17,975 KPs, comprising female sex workers (FSWs), gay men and other men who have sex with men (MSM), transgender persons (TG), and people who inject drugs (PWID) [25]. The participants were recruited via a multistage sampling approach. Additional details about the survey methodology are available in the report [25]. The analytic sample included all 17,975 KPs.

### 2.2. Measures and Operational Definitions

We measured discriminatory attitudes towards PLHIV from the following questions in the survey: (i) Care for PLHIV (yes/no): If a relative of yours became sick with AIDS, would you be willing to care for him/her in your household? (ii) Buy food from PLHIV (yes/no): If you knew that a shopkeeper or food seller had HIV, would you buy food from him/her? (iii) Work with PLHIV (yes/no): If a colleague in your place of work has HIV, would you be willing to continue working with him/her? (v) Share a meal with PLHIV (yes/no): Would you be willing to share a meal (eat from the same plate) with a person you knew had HIV or AIDS? and (vi) Quarantine PLHIV (yes/no): Do you think people infected with HIV or AIDS should be quarantined (that is, physically separated from the rest of society)? We excluded the question: “If a member of your family became ill with HIV, the virus that causes AIDS, would you want it to remain a secret?” from our analysis because both positive and negative responses to the question can be interpreted as stigmatizing [26]. We defined discriminatory attitudes towards PLHIV as negative responses to providing care for PLHIV, buying food from PLHIV, working with PLHIV, and sharing a meal with PLHIV and positive responses to quarantining PLHIV.

The other variables included in the analysis were: (1) Consistent condom use (yes/no): frequency of condom use with any sexual partners in the last 12 months. We classified “every time” as “yes” and “almost every time,” “sometimes,” and “never” as “no.” (2) Perceived risk of HIV (yes/no): Felt at risk for infection with HIV. (3) Exposure to HIV information (yes/no): Receipt of any information or education about HIV, AIDS, sexually transmitted diseases, or condoms within the past 12 months. (4) Comprehensive HIV knowledge (yes/no): Correctly identifying that a healthy-looking person can be infected with HIV, three methods of HIV transmission (sharing of needles, blood transfusion, and mother-to-child transmission) and rejection of one misconception about HIV transmission (using the same plate/utensils with someone who is infected). Those with incorrect responses to any of the questions were classified as “no”. (5) HIV status (positive/negative/unknown): Self-reported HIV status. (6) Sociodemographic variables: age (15–24/≥ 25 years), educational attainment (none/primary/secondary/tertiary), religion (Christianity/Islam/others or none), marital status (single/currently married/formerly married [separated, divorced, or widowed]), employment status (employed/unemployed/out of the labor force [students or retirees]), and geopolitical zone (South East/South South/South West/North Central/North East/North West).

### 2.3. Data Analysis

We conducted weighted descriptive statistics to summarize the data. Latent class analysis (LCA) was used to determine the patterns of discriminatory attitudes [27, 28]. The five questions on discriminatory attitudes were used in the LCA to determine the classes. Starting with two classes, we incrementally examined LCA models up to five classes. The models were compared using the Akaike's Information Criteria (AIC), Bayesian Information Criteria (BIC), and entropy, with lower AIC and BIC values and higher entropy values indicating better model fit. In addition, we also considered the parsimony of the LCA models and the interpretability of the latent classes in our selection [29]. We estimated the predicted probabilities of the classes for each KP characteristic while holding all other characteristics in the model at their means. Missing data was handled through listwise deletion. The data analysis was performed with Stata 18.

## 3. Results

### 3.1. Description of the Study Population

From the 17, 975 respondents, FSW represented 27.7%, MSM 24.5%, PWID 24.6%, and TG 23.3% (Table 1). Most of the participants were aged 25 years or older (59.5%), identified as Christians (74.7%), single (79.6%), and employed (47.5%). About 87% had at least a secondary education and 63.5% resided in the southern zones. Sixty percent of the KPs reported exposure to HIV information, while 55.4% had comprehensive HIV knowledge. Approximately 89% of the respondents self-reported a negative HIV status. About 13.5%, 29.7%, 15.8%, and 27.9% of the participants reported that they would not care for, buy food from, work with, and share a meal with PLHIV, respectively. Additionally, 16.3% of the participants believed that PLHIV should be quarantined (Table 1).

### 3.2. LCA

The 3- and 4-class models showed the best fit based on the fit statistics. Although the 4-class model had slightly lower AIC and BIC values than the 3-class model (Table 2), we selected the 3-class model because it presented a more meaningful interpretation. Additionally, the 3-class model had a higher entropy level, suggesting a better class separation. The latent class membership and item-response probabilities for the 3-class model are shown in Table 3. Class 1 (low discriminatory attitude) comprised 65% of the study population. This class had low probabilities of each of the five discriminatory attitude items (< 50%): would not work with PLHIV (0.6%), would not share a meal with PLHIV (0.8%), would not care for PLHIV (2.9%), and would want PLHIV to be quarantined (11.9%) (Table 3). Class 2 (moderate discriminatory attitude) represented 23% of the study population. This class had high probabilities (50%) for two of the five discriminatory attitude items: would not share a meal with (60.8%) and would not buy food from PLHIV (76%). Class 3 (high discriminatory attitude) comprised 11% of the study population. This class had high probabilities (> 50%) for four of the five discriminatory attitude items: would not care for PLHIV (74.1%), would not work with PLHIV (91.8%), would not share a meal with PLHIV (94.8%), and 99.0% would not buy food from PLHIV (99.0%) (Table 3).

### 3.3. Adjusted Predicted Probabilities of Discriminatory Attitudes Towards PLHIV Class Membership

The adjusted predicted probabilities of discriminatory attitudes towards PLHIV class membership for each of the KP characteristics are presented in Table 4. Compared with others, the predicted probability of high discriminatory attitude was higher in an average KP who injects drug (27.5%), young (23.7%), with primary education (24.1%), Muslim (26.9%), formerly married (23.9%), out of the labor force (21.3%), in the South East (39.7%), with no risk perception (24%), with no comprehensive HIV knowledge (25.1%), not exposed to HIV message (25.3%), and with unknown HIV status (42.0%).

## 4. Discussion

Using the 2020 IBBSS, we examined the discriminatory attitudes towards PLHIV among KPs in Nigeria. Three latent classes were identified: Low discriminatory attitude (65%), moderate discriminatory attitude (23%), and high discriminatory attitude (11%). The predicted probability of high discriminatory attitude was highest in KPs with unknown HIV status (42%) followed by those residing in the South East (39.7%) and injection drug users (27.5%). Our results highlight the need to pay close attention to and address discriminatory attitudes among KPs.

While discrimination against PLHIV has been widely reported among the general population, this study demonstrates that KPs can also be important perpetrators of such discrimination. However, the prevalence of discriminatory attitudes among KPs appears to be lower than in the general population. For example, a previous study among the general population in Nigeria [12] reported that 50% of the respondents would not buy vegetables from a vendor living with HIV, and 30% would not care for relatives with AIDS. In comparison, our study found that 30% of KPs would not buy food from PLHIV and 13.5% that would not care for them. Perceived contagiousness of HIV may be one of the several reasons why KPs discriminate against PLHIV. Other reasons why HIV is stigmatized include the manifestation of the disease, mode of transmission, lack of cure, and nature of people at increased risk [4, 30, 31].

Compared with other typologies, the predicted probability of high discriminatory attitude was higher in an average injection drug user and FSW. The explanation for these differences is not apparent and warrant further qualitative investigation. However, the relatively lower HIV prevalence among PWID and FSW may partially explain their higher levels of discriminatory attitude, as they may be less likely to be infected with HIV or to have PLHIV within their social networks. Notably, more negative attitudes towards PLHIV have been reported in settings with lower HIV prevalence [32].

Consistent with previous studies conducted in the general population [9–11], our findings highlight the inverse relationship between HIV knowledge and discriminatory attitudes towards PLHIV. Specifically, we found that an average KP without comprehensive HIV knowledge is nearly twice as likely as an average KP with comprehensive HIV knowledge to have a high discriminatory attitude towards PLHIV. The importance of HIV knowledge is further supported by the observed lower predicted probabilities of low discriminatory attitude in more educated KPs or KPs exposed to HIV messages. Comprehensive knowledge may help equip KPs with accurate information and reduce unwarranted fears about HIV, thereby reducing discriminatory attitudes.

The results also indicated that HIV testing is associated with lower levels of discriminatory attitudes towards PLHIV, with KPs who are aware of their HIV status being less likely to have high discriminatory attitudes. Similar findings have been reported in the general population [9]. HIV testing provides a critical opportunity to provide accurate information, dispel myths, and reduce misconceptions that may lead to discriminatory behaviors. However, despite the significant progress, HIV testing rates among KPs remain suboptimal [33]. As expected, the probability of low discriminatory attitude was higher among KPs living with HIV, suggesting that engaging them in peer-led treatment interventions may be particularly appropriate. KPs living with HIV are likely to be more empathetic and less judgmental towards others living with HIV.

The associations observed between discriminatory attitudes and both geographical regions (proxy for ethnicity) and religion point to the significant role of culture in shaping HIV-related stigma and discrimination. In some settings, HIV is still perceived as a consequence of sinful or immoral behaviors [34, 35]. Notably, ethnicity has been identified as a stronger predictor of discrimination than religion [36]. Nonetheless, consistent with our findings, discriminatory attitudes have been reported to be higher among Muslims and other religions compared with Christians [36]. With regard to ethnicity, the predicted probability of high discriminatory attitude was 39.5% in the Southeast (dominated by the Igbo ethnic group) region relative to a range of 9.7%–25.5% in other regions. This aligns with findings from a previous study in the general population, which reported higher discriminatory behavior among Igbos, compared with Hausa, Yoruba, and Fulani ethnic groups [37]. These results highlight the need for community-specific interventions to address HIV-related discriminatory attitudes effectively.

Although important progress has been made, significant gaps remain in stigma-reduction research [38–41]. Interventions to reduce HIV-related stigma have been broadly categorized into six types: (1) information-based approaches, (2) skills building, (3) counselling/support, (4) contact with affected groups, (5) structural, and (6) biomedical [38, 42]. Stigma-reduction interventions are likely to be most effective if culturally appropriate and targeted at multiple levels [38, 43]. In Nigeria, one notable structural intervention efforts to reducing stigma has been the enactment of the HIV antidiscrimination law [44]. However, evidence regarding the effectiveness of such laws in reducing stigma remains limited. The impact of HIV antidiscrimination laws partly depends on whether PLHIV are aware of their rights and the means to enforce them [38].

To the best of our knowledge, this study is among the first to report on discriminatory attitudes towards PLHIV among KPs in SSA. The use of nationally representative data across Nigeria's six geopolitical zones enhances the generalizability of the findings. While the outcome measures are commonly used for assessing discriminatory attitudes towards PLHIV, they are based on hypothetical scenarios and self-reported responses, which may be subject to social desirability bias. Additionally, the measures assessed attitudes toward PLHIV in general and may not fully capture attitudes specifics to KPs living HIV. While these findings are suggestive, further research is needed to explore how KPs perceive PLHIV within their own social networks.

## 5. Conclusion

Discriminatory attitudes towards PLHIV are prevalent among KPs in Nigeria, although they appear to occur at a lower rate compared with the general population. Addressing these attitudes among KPs is essential, particularly in settings where they may be engaged in social network-based prevention, treatment, and care services. We recommend that such programs incorporate prescreening for discriminatory attitudes and negative perceptions about HIV. Stigma and discrimination reduction efforts in Nigeria and similar settings should recognize that KPs can be both victims and perpetrators of HIV-related discrimination and design interventions accordingly.

## Figures and Tables

**Table 1:** Characteristics of the study population and discriminatory attitudes towards PLHIV, IBBSS 2020 (*N* = 17,975).

Characteristics	N (%)
Typology	
FSW	4974 (27.7)
MSM	4397 (24.5)
PWID	4414 (24.6)
TG	4190 (23.3)
Age (years)	
15–24	7263 (40.5)
≥ 25	10,711 (59.5)
Educational attainment	
None^∗^	704 (3.9)
Primary	1716 (9.5)
Secondary	11,323 (63.0)
Tertiary	4232 (23.5)
Religion	
Christianity	13,318 (74.7)
Islam	3912 (21.9)
Others or none	593 (3.3)
Missing	152
Marital status	
Single	14,303 (79.6)
Currently married	1471 (8.2)
Formerly married^∗∗^	2201 (12.2)
Employment status	
Employed	8450 (47.5)
Unemployed	6773 (38.0)
Out of the labor force^∗∗∗^	2579 (14.5)
Missing	172
Geopolitical zone	
South East	1360 (7.6)
South South	4288 (23.9)
South West	5745 (32.0)
North Central	3899 (21.7)
North East	519 (2.9)
North West	2163 (12.0)
Risk perception	
No	9468 (60.8)
Yes	6111 (39.2)
Missing	2396
Comprehensive HIV knowledge	
No	7931 (44.6)
Yes	9833 (55.4)
Missing	211
Exposure to HIV message	
No	6835 (39.9)
Yes	10,296 (60.1)
Missing	843
HIV status	
Negative	12,638 (88.9)
Positive	1009 (7.1)
Unknown	5,71 (4.0)
Missing	3757
Care for PLHIV	
No	2252 (13.5)
Yes	14,419 (86.5)
Missing	1303
Buy food from PLHIV	
No	4980 (29.7)
Yes	11,774 (70.3)
Missing	1221
Work with PLHIV	
No	2647 (15.8)
Yes	14,116 (84.2)
Missing	1213
Share a meal with PLHIV	
No	4662 (27.9)
Yes	12,069 (72.1)
Missing	1244
Quarantine PLHIV	
No	13,708 (83.7)
Yes	2661 (16.3)
Missing	1605

^∗^No formal education.

^∗∗^Separated, divorced, or widowed.

^∗∗∗^Students or retirees.

**Table 2:** Model fit indices for the latent classes.

	Df	AIC	BIC	Entropy
2-class	11	70,609.08	70,694.55	0.847
3-class	17	70,019.23	70,151.32	0.742
4-class	22	69,964.43	70,143.14	0.690
5-class	25	669,970.32	70,180.12	0.488

**Table 3:** Latent class membership and item-response probabilities for the 3-class model of discriminatory attitudes towards PLHIV among KPs.

	Class 1: Low discriminatory attitude	Class 2: Moderate discriminatory attitude	Class 3: High discriminatory attitude
	Probability (95% CI)	Probability (95% CI)	Probability (95% CI)
Class membership	0.652 (0.618–0.683)	0.234 (0.215–0.255)	0.114 (0.096–0.135)
Item response^∗^			
Care for PLHIV	0.029 (0.025–0.034)	0.175 (0.141–0.216)	0.741 (0.677–0.796)
Buy food from PLHIV	0.039 (0.023–0.065)	0.760 (0.688–0.820)	0.990 (0.969–0.997)
Work with PLHIV	0.006 (0.003–0.012)	0.279 (0.219–0.348)	0.918 (0.863–0.951)
Share a meal with PLHIV	0.080 (0.068–0.093)	0.608 (0.538–0.674)	0.948 (0.922–966)
Quarantine PLHIV	0.119 (0.112–0.126)	0.234 (0.207–0.263)	0.496 (0.461–0.531)

^∗^Negative responses to providing care for PLHIV, buying food from PLHIV, working with PLHIV, sharing a meal with PLHIV, and positive response to quarantining PLHIV.

**Table 4:** Adjusted predicted probabilities of discriminatory attitudes towards PLHIV class membership for KP characteristics.

	Class 1: Low discriminatory attitude	Class 2: Moderate discriminatory attitude	Class 3: High discriminatory attitude
	Predicted probability (95% CI)	Predicted probability (95% CI)	Predicted probability (95% CI)
Typology			
FSW	0.715 (0.692–0.739)	0.017 (0.007–0.026)	0.268 (0.246–0.290)
MSM	0.835 (0.816–0.855)	0.016 (0.007–0.025)	0.149 (0.132–0.166)
PWID	0.685 (0.645–0.725)	0.041 (0.015–0.066)	0.275 (0.249–0.301)
TG	0.869 (0.852–0.887)	0.014 (0.006–0.022)	0.117 (0.101–0.132)
Age (years)			
15–24	0.741 (0.717–0.764)	0.022 (0.010–0.034)	0.237 (0.218–0.257)
25–34	0.802 (0.785–0.820)	0.018 (0.07–0.029)	0.180 (0.166–0.193)
Educational attainment			
None	0.766 (0.706–0.826)	0.056 (0.024–0.093)	0.177 (0.132–0.223)
Primary	0.740 (0.702–0.777)	0.019 (0.006–0.032)	0.241 (0.207–0.275)
Secondary	0.765 (0.747–0.783)	0.018 (0.008–0.028)	0.217 (0.203–0.231)
Tertiary	0.826 (0.803–0.848)	0.021 (0.008–0.033)	0.153 (0.136–0.171)
Religion			
Christianity	0.803 (0.785–0.821)	0.021 (0.009–0.032)	0.176 (0.163–0.190)
Islam	0.716 (0.688–0.743)	0.016 (0.006–0.025)	0.269 (0.243–0.294)
Others or none	0.761 (0.690–0.831)	0.044 (0.011–0.077)	0.195 (0.136–0.255)
Marital status			
Single	0.788 (0.770–0.806)	0.019 (0.009–0.030)	0.192 (0.179–0.205)
Currently married	0.769 (0.734–0.803)	0.020 (0.007–0.033)	0.211 (0.180–0.242)
Formerly married	0.740 (0.708–0.772)	0.021 (0.009–0.034)	0.239 (0.210–0.268)
Employment status			
Employed	0.772 (0.753–0.790)	0.164 (0.007–0.026)	0.212 (0.196–0.227)
Unemployed	0.795 (0.773–0.817)	0.025 (0.011–0.038)	0.180 (0.164–0.196)
Out of the labor force	0.767 (0.734–0.799)	0.020 (0.007–0.033)	0.213 (0.184–0.243)
Geopolitical zone			
South East	0.547 (0.503–0.591)	0.055 (0.029–0.083)	0.397 (0.362–0.432)
South South	0.748 (0.724–0.773)	0.003 (< 0.001–0.005)	0.249 (0.225–0.273)
South West	0.737 (0.714–0.761)	0.008 (0.002–0.014)	0.255 (0.233–0.277)
North Central	0.856 (0.834–0.878)	0.021 (0.009–0.033)	0.123 (0.104–0.141)
North East	0.851 (0.818–0.883)	0.052 (0.025–0.079)	0.097 (0.080–0.114)
North West	0.792 (0.753–0.831)	0.057 (0.025–0.089)	0.151 (0.130–0.172)
Risk perception			
No	0.745 (0.728–0.761)	0.011 (0.005–0.018)	0.244 (0.229–0.259)
Yes	0.811 (0.784–0.838)	0.040 (0.019–0.062)	0.148 (0.134–0.163)
Comprehensive HIV knowledge			
No	0.652 (0.612–0.693)	0.097 (0.062–0.131)	0.251 (0.232–0.270)
Yes	0.836 (0.823–0.849)	0.006 (0.001–0.010)	0.158 (0.146–0.170)
Exposure to HIV message			
No	0.727 (0.704–0.751)	0.020 (0.008–0.031)	0.253 (0.234–0.273)
Yes	0.803 (0.786–0.820)	0.020 (0.009–0.030)	0.177 (0.164–0.180)
HIV status			
Negative	0.775 (0.758–0.792)	0.019 (0.009–0.029)	0.206 (0.193–0.218)
Positive	0.903 (0.872–0.934)	0.025 (0.007–0.043)	0.071 (0.049–0.096)
Unknown	0.559 (0.495–0.622)	0.021 (0.005–0.037)	0.420 (0.358–0.480)

## Data Availability

The data that support the findings of this study are available on request from the National Agency for the Control of AIDS, Nigeria.
